# Phosphoregulation of the Plant Cellulose Synthase Complex and Cellulose Synthase-Like Proteins

**DOI:** 10.3390/plants7030052

**Published:** 2018-06-29

**Authors:** Tori L. Speicher, Patrick Ziqiang Li, Ian S. Wallace

**Affiliations:** Department of Biochemistry and Molecular Biology, University of Nevada, Reno, NV 89557, USA; tspeicher@nevada.unr.edu (T.L.S.); patrick.lee@nevada.unr.edu (P.Z.L.)

**Keywords:** cellulose synthase complex, plant cell wall, protein kinases, cellulose biosynthesis, cellulose synthase-like

## Abstract

Cellulose, the most abundant biopolymer on the planet, is synthesized at the plasma membrane of plant cells by the cellulose synthase complex (CSC). Cellulose is the primary load-bearing polysaccharide of plant cell walls and enables cell walls to maintain cellular shape and rigidity. The CSC is comprised of functionally distinct cellulose synthase A (CESA) proteins, which are responsible for synthesizing cellulose, and additional accessory proteins. Moreover, CESA-like (CSL) proteins are proposed to synthesize other essential non-cellulosic polysaccharides that comprise plant cell walls. The deposition of cell-wall polysaccharides is dynamically regulated in response to a variety of developmental and environmental stimuli, and post-translational phosphorylation has been proposed as one mechanism to mediate this dynamic regulation. In this review, we discuss CSC composition, the dynamics of CSCs in vivo, critical studies that highlight the post-translational control of CESAs and CSLs, and the receptor kinases implicated in plant cell-wall biosynthesis. Furthermore, we highlight the emerging importance of post-translational phosphorylation-based regulation of CSCs on the basis of current knowledge in the field.

## 1. Introduction

Plant cell walls are complex polysaccharide-rich extracellular matrices that surround all plant cells and critically influence basic cellular growth processes, such as cell expansion, cell division, and the acquisition of cell shape [1,2,3]. Fundamentally, the cell wall provides mechanical support to aid upright growth; forms a protective layer that surrounds all plant cells; dictates plant cell shape [4]; and regulates key physiological processes, such as stomatal opening [5], reproduction [6], and selective nutrient uptake [7].

Plant cell walls can be largely grouped into primary cell walls (PCWs), which surround all growing cells, and secondary cell walls (SCWs), which mechanically support specialized non-expanding cells, such as xylem in vascular tissues [8]. The precise chemical composition of the cell wall changes during growth and development or in response to environmental stresses [9], but PCWs typically consist of three polysaccharide networks: cellulose, neutral hemicelluloses, and acidic pectins. Furthermore, SCWs contain polyphenolic lignin polymers as an additional component of the cell-wall network [10,11]. Cellulose is synthesized at the plasma membrane and consists of 18–24 *β*-(1→4)-d-glucan chains that are organized into paracrystalline microfibrils [1,2,3,12,13], which provide the major structural rigidity of the cell-wall matrix [1,13,14]. Hemicelluloses and pectins are structurally and chemically diverse polysaccharides that are synthesized in the endomembrane system before they are transported to the extracellular matrix [15]. Once synthesized, cellulose, hemicelluloses, and pectins are deposited into the apoplast, where they dynamically work in concert to form functional cell walls.

Plant growth and development is a consecutive process consisting of both cell-division and cell-expansion events, and cellulose deposition plays key roles in these processes [16]. During cytokinesis, a new cell wall must be synthesized, and cellulose is deposited at the developing cell plate between two mature daughter cells [17,18,19]. Genetic mutations impacting cellulose production often cause incomplete cell-plate formation in developing embryos as well as in root and shoot meristems, highlighting the importance of cellulose deposition during cytokinesis [20,21,22]. Cellulose biosynthesis also plays a key role in cell expansion, whereby cellulose deposition typically occurs perpendicular to the axis of cell growth and elongation [23]. In highly anisotropic cells, such as root hairs or hypocotyl epidermal cells, the cellulose microfibril tensile strength is proposed to shape anisotropic cell expansion by spatially resisting the internally generated osmotic force that is required for turgor-mediated cell growth. As a result, cellulose biosynthetic mutants often exhibit compromised cell elongation, cell swelling, cell bursting, and irregular cell-shape phenotypes [13].

Cell-wall biosynthesis also plays an important role during the management of abiotic and biotic stresses. Cellulose is often targeted by microbial glycosyl hydrolases, such as cellulases, which are secreted from pathogens [24]. Under osmotic and salinity stresses, cellulose biosynthesis temporarily ceases, suggesting that the reprogramming of cellulose biosynthesis is necessary under abiotic stresses and that such responses might reinforce plants with better abiotic stress adaptations, which is supported by the observation that many cellulose biosynthetic mutants are more sensitive to abiotic stresses [22,25,26,27,28].

Many outstanding recent reviews have summarized the current progress in cell-wall biosynthesis [29,30], biochemical and cell biological perspectives of cellulose biosynthesis [13,31], and cell-wall signaling [32]. Here, we mainly focus on the post-translational regulation of cellulose biosynthesis and the biosynthetic regulation of other cell-wall polymers.

## 2. The Cellulose Synthase Complex

### 2.1. Composition of the Plant Cellulose Synthase Complex

**Figure 1:**
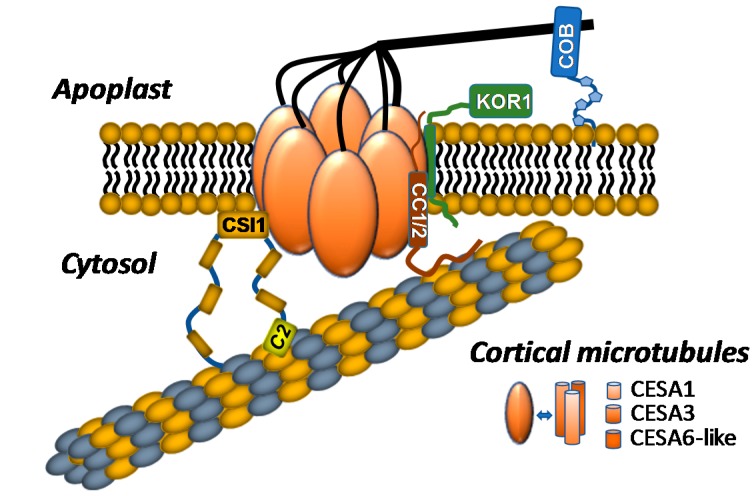
The current model of the plant cellulose synthase complex (CSC) and CSC-associated proteins in vivo is shown. CSC forms hexagonal rosettes with 3 subunits of cellulose synthase A (CESA) comprising 1 rosette lobe, resulting in 18 CESAs within each CSC. CSC-associated proteins include cellulose synthase interactive protein 1 (CSI1), companion of cellulose synthase 1/2 (CC1/2), KORRIGAN1 (KOR1), and COBRA. CSI1 bridges CSCs to the cortical microtubules (MTs) through binding of its C2 domain and facilitates bidirectional movement of CSCs through the plasma membrane. CC1 and CC2 bind to MTs via the CC1/2 N-terminal domain, which is needed for maintaining CSC activity and MT dynamics under salt stress. KOR1 is an integral component of the CSC and possibly regulates cellulose biosynthesis through its endo-1,4-ß-d-glucanase activity. COBRA, a glycosylphosphatidylinositol (GPI)-anchored protein, is located in the extracellular matrix and regulates CSC activity, cellulose organization, and interaction with other cell-wall components.

Cellulose is synthesized at the plasma membrane by a large multiprotein complex known as the cellulose synthase complex (CSC) (Figure 1). CSCs were first observed by freeze-fracture electron microscopy as lobed 25–30 nm complexes exhibiting six-fold symmetry [33,34]. These complexes were observed at the termini of cellulose microfibrils and were named “terminal complexes”; they were later renamed “rosettes” because of their flower-shaped architecture. In higher plants, CSCs contain multiple non-redundant cellulose synthase A (CESA) proteins that serve as CSC catalytic subunits [35,36]. CESAs polymerize *β*-(1→4)-linked glucan chains that are deposited into the apoplast using UDP-glucose as a donor substrate. These proteins exhibit a conserved domain architecture consisting of an extended N-terminal domain that contains a zinc finger motif, eight transmembrane domains, and a large central catalytic domain that contains conserved amino acid signatures of family 2 processive glycosyltransferases. The *Arabidopsis* genome encodes 10 *CESA* genes (*CESA1–10*), with *CESA1*, *CESA3*, and *CESA6*-like (*CESA2*, *CESA5*, *CESA6*, and *CESA9*) genes involved in PCW cellulose biosynthesis, and *CESA4*, *CESA7*, and *CESA8* involved in SCW cellulose biosynthesis [13,37,38,39]. Immunoprecipitation and mass spectrometry have confirmed that *CESA1*, *CESA3*, and *CESA6* are present at equimolar ratios in the PCW CSC, and the same observation holds true for the SCW protein subunits *CESA4*, *CESA7*, and *CESA8* [12,40]. The exact number of CESAs in one CSC is still under debate, but current models based on cryo-electron microscopy and computational modeling suggest that CSCs contain a hexamer of CESA trimers, where each lobe of the rosette comprises three CESA subunits [41,42].

Once they arrive at the plasma membrane, CSCs are activated and begin producing cellulose by a mechanism that is currently unclear. Recent *in vitro* enzymology data indicates that CESAs are capable of producing cellulose in the presence of UDP-glucose, suggesting that no additional factors are necessary for CESA activity [35]. However, cellulose biosynthesis is much more complex *in vivo*. Plasma-membrane-localized CSCs form small motile particles that move with a constant velocity of approximately 250 nm/min, and these complexes are guided along cortical microtubule (MT) trajectories [23,43,44,45]. On the basis of genetic, inhibitor, and computational studies, it has been postulated that cellulose polymerization into the apoplast is responsible for CSC motility [23,46,47,48].

### 2.2. Proteins Associated with the CSC

In addition to CESAs, numerous CSC-associated subunits have been identified, primarily through transcriptional co-expression analyses, through genetic analyses of mutants compromised in cellulose biosynthesis, or through yeast two-hybrid screens [45,49,50,51]. Among these CSC-associated proteins are KORRIGAN1 (KOR1), cellulose synthase interactive protein 1 (CSI1), the companion of cellulose synthase (CC) proteins, and the glycosylphosphatidylinositol (GPI)-anchored protein COBRA (Figure 1).

KOR1 is a *β*-(1→4)-endoglucanase that directly interacts with CESA subunits and colocalizes with plasma-membrane CSCs [20,52,53]; *kor1* mutants exhibit phenotypic deficiencies consistent with cellulose biosynthetic mutants, including epidermal cell swelling, cellulose deficiency, and reduced root elongation [20,52,53,54,55,56]. CSC motility is also impaired in *kor1* mutants, suggesting that KOR1 endoglucanase activity stimulates cellulose biosynthesis [48,53]. Taken together, these observations indicate that KOR1 is an integral component of CSCs that stimulates cellulose biosynthesis through a currently unknown mechanism.

CSI1 is a 2150 amino acid armadillo repeat/C2-domain-containing protein that was identified through a yeast two-hybrid screen for proteins that directly interact with the *Arabidopsis* CESA6 catalytic domain; *csi1* mutants negatively impact CSC motility at the plasma membrane, exhibit cell elongation defects, and display reduced cellulose content in hypocotyls and roots [45]. Live-cell imaging analyses and in vitro MT binding data indicate that CSI1 physically interacts with both CESAs and cortical MTs [22,45,57]. Additionally, CSCs lose their ability to track along cortical MTs in *csi1* mutants, indicating that CSI1 guides CSCs along cortical MT arrays, thus serving as a functional link between MTs and CSCs [57]. Later studies demonstrated that deletion of the CSI1 C2 domain resulted in mislocalization of CSI1 to the cytosol, indicating that the C2 domain is crucial for mediating CSI1 localization and CSC interaction [22]. The C2 domain is also required for MT binding *in vivo* and *in vitro* [58]. Recently, CSI1 was shown to aid de novo CSC secretion through cooperation with the exocyst complex, and the plant-specific protein PATROL1. Live-cell imaging suggests that CSI1 marks the docking site for CSC-containing vesicles by mediating their interaction with MTs [49]. These observations indicate that CSI1 plays multiple roles in the delivery of CSCs to the plasma membrane and in the cytoskeleton-assisted guidance of active CSCs.

The companion of cellulose synthase (CC) proteins are encoded by a group of four genes in the *Arabidopsis* genome. CC proteins were also recently demonstrated to directly interact with CSCs and MTs. Abiotic stresses cause the depolymerization of MTs and internalization of active plasma-membrane-localized CSCs, but CC proteins mediate the return of CSCs to the plasma membrane after the stress is imposed [28]. Live-cell imaging demonstrates that CC proteins facilitate trafficking of CSCs back to the plasma membrane after salt stress, suggesting that these proteins serve as stress-dependent trafficking chaperones for the CSC [28]. CC proteins are plant-specific proteins that contain an extended N-terminal domain that is localized to the cytosol followed by a single transmembrane domain and a putative extracellular domain of unknown function [28]. Interestingly, the N-terminal domain of CC1 was sufficient to mediate MT binding and bundling *in vitro* as well as complement the salt-stress-induced phenotype of the *cc1*;*cc2* double mutant, indicating that this domain may play an important role in CSC trafficking under abiotic stress conditions [28].

COBRA, a GPI-anchored protein localized to the plasma membrane in *Arabidopsis*, also plays a key role in determining the orientation of epidermal cell expansion [59]. COBRA-like proteins interact with glucans *in vitro*, suggesting that this protein may play a role in glucan biosynthesis or aggregation. *cobra* mutants display irregularly expanding roots [60] and cellulose deficiencies, implicating this protein in the process of cellulose biosynthesis. Interestingly, a subgroup of COBRA-like genes have been associated with cellulose biosynthesis in a tissue-specific manner [61]. For example, the *COBRA-LIKE 2* gene is involved in cellulose deposition specifically in seed coat mucilage secretory cells [62], and the rice COBRA-Like *BRITTLE CULM1* [63] gene plays a role in cellulose assembly through interactions with cellulose microfibrils [63]. Despite this information, the precise functions of COBRA and COBRA-like proteins remain unclear.

### 2.3. The In Vivo Dynamics of the Plant CSC

Live-cell microscopy has revealed that CSC subcellular localization and dynamics are complex [23,43,44]. CSC subunits are localized to the Golgi apparatus, where complex assembly is assisted by STELLO proteins [64] before CSCs are trafficked to the plasma membrane via post-Golgi compartments called small CESA compartments (SmaCC) [58] or MT-associated cellulose synthase compartments (MASCs) [43,44]. CSC organization and protein complex composition are poorly understood in Golgi and SmaCC/MASCs [13]. CSCs are transported to the plasma membrane via SmaCC/MASCs in a cytoskeleton-assisted manner [43,44]. SmaCC/MASC vesicles can carry one or two CSCs and subsequently deliver the cargo to the plasma membrane. Actin controls the cytosolic distribution of these vesicles, and cortical MTs position SmaCC/MASCs for delivery to the plasma membrane [43,44]. Additionally, the chemical and genetic disruption of actin organization inhibits SmaCC/MASC movement in the cytosol and overall exocytic rates, which results in cellulose deficiencies [44,65,66]. In the subcortical region, SmaCCs exhibit fast, erratic movement along actin. The disruption of actin or MTs results in an increased number of SmaCC/MASCs associated with the other cytoskeletal array and dynamic interaction of cortical actin and MTs in interphase plant cells, suggesting an exchange of SmaCC/MASCs between the two cytoskeletal arrays [44,65].

## 3. Phosphoregulation of CSC and CESA-Like Proteins

### 3.1. Identification and Characterization of CSC-Associated Phosphorylation Events

**Figure 2:**
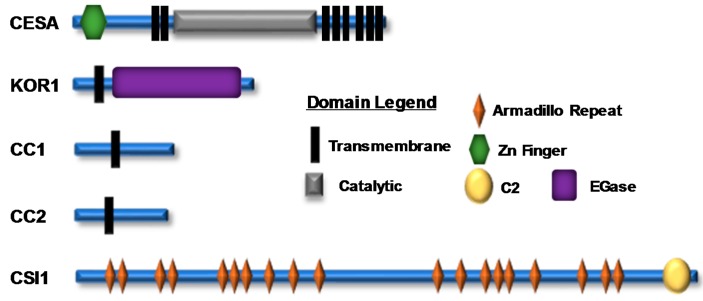
A representation of cellulose synthase A (CESA) and cellulose synthase complex (CSC)-associated proteins; their domain structure and location are shown. There are numerous experimentally identified phosphorylation sites within the N-terminal and catalytic domains of CESA proteins and within various CSC accessory proteins (Supplementary Table S1). The icons corresponding to each domain are shown in the domain legend.

Plant cell-wall biosynthesis changes in response to many environmental and cellular cues, which can negatively or positively impact the deposition of newly synthesized cell-wall material, suggesting that this biosynthetic process is intricately controlled at multiple regulatory levels. Considering these observations, it is important to note that numerous large-scale phosphoproteomic surveys have identified a myriad of post-translational phosphorylation sites within CSC subunits [67,68,69]. For example, CESA proteins are phosphorylated at numerous positions throughout their N-terminal domains or in the hypervariable region of their large central catalytic loop (Figure 2). Additionally, CSC accessory proteins, such as CSI1, KOR1, and the CC proteins, are extensively phosphorylated throughout their cytosolic domains. These observations suggest that post-translational phosphorylation plays an important role in governing plant cellulose biosynthesis (Figure 2) [67,68,69,70,71].

**Figure 3:**
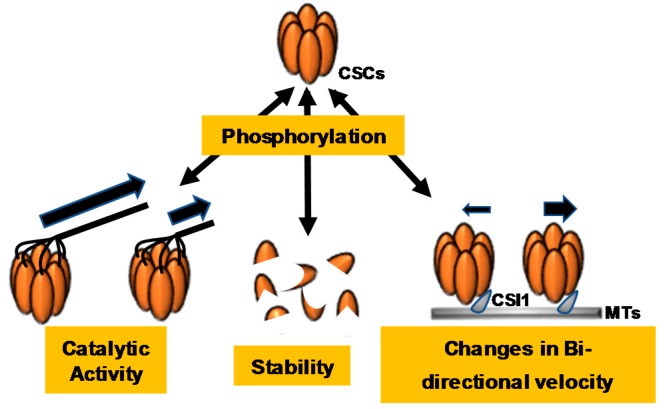
Cellulose synthase complex (CSC) phosphorylation events dynamically regulate CSC catalytic activity, stability, and bidirectionality. Cellulose synthase A7 (CESA7) phosphorylation leads to rapid degradation of the protein, indicating that CESA7 phosphorylation regulates protein stability. CESA1, CESA3, and CESA5 phosphorylation regulate changes in CSC velocities and constant motility, indicating that phosphorylation of these CESAs regulates CSC catalytic activity and bidirectional motility.

The identification of numerous CSC protein phosphorylation sites has led to the study of CSC phosphoregulation and its implications for CSC catalytic function. Many of the experimentally supported phosphorylation sites within CESA proteins have been mutated to either phosphonull or phosphomimic residues, and these mutant enzymes have been examined for changes in phenotype or changes in CSC dynamics *in vivo* (Supplementary Table S1 and Figure 3). For example, all experimentally supported phosphorylation sites within the CESA1 amino-terminal and catalytic domains were mutated to either phosphonull (A) or phosphomimic (E) residues, and these constructs were expressed in the temperature-sensitive *cesa1^rsw1^* mutant background; *cesa1^rsw1^* lines expressing the phosphonull mutations CESA1^S686A^ and CESA1^S688A^ exhibited shorter etiolated hypocotyl lengths, while the phosphomimic mutants CESA1^S686E^ and CESA1^S688E^ displayed a longer root length when compared to the *rsw1* line expressing CESA1^wt^, suggesting that phosphorylation at these sites within the catalytic domain promotes CSC catalytic activity [72]. Additionally, four phosphorylation sites within the amino-terminal domain of CESA1 (CESA1 S162, T165, T166, and S167) were also examined. CESA1^S162E^, CESA1^T165E^, CESA1^T166E^, and CESA1^S167E^ all exhibited reduced root and hypocotyl growth, while CESA1^T166A^ displayed increased root and hypocotyl growth [72]. The CSC *in vivo* motility behavior of each phosphorylation site mutant was also examined by live-cell microscopy. Wild-type CSCs move bidirectionally along cortical MTs with similar velocities [23]. Interestingly, the authors found that impaired cell expansion in these phosphorylation site mutants positively correlated with both reduced bidirectional velocities of CSCs moving at the plasma membrane and reduced crystalline cellulose contents [72], suggesting that post-translational phosphorylation of some sites within CESAs could potentially regulate CESA–MT association and overall crystalline cellulose output. Interestingly, many of the bidirectional velocity effects were eliminated in the presence of the MT depolymerizing agent oryzalin [72], suggesting that MTs are necessary for this phenomenon.

Similar experiments were performed to investigate the role of CESA3 phosphorylation sites in anisotropic cell expansion. The CESA3^S211A^ phosphonull mutant exhibited 28% and 47% reductions in primary root and hypocotyl length, whereas the CESA3^S211E^ phosphomimic mutant showed only a minor decrease in hypocotyl length and no effect on primary root length compared to the CESA3^wt^ complementary line. These results indicate that phosphorylation at CESA3^S211^ positively regulates CESA3 motility [73]. Interestingly, an opposite effect was observed in the CESA3^T212A^ and CESA3^T212E^ mutants. CESA3^T212A^ displayed no obvious change in etiolated hypocotyl or primary root morphology, while the CESA3^T212E^ mutant exhibited a 43% decrease in hypocotyl length. Therefore, the phosphorylation events at CESA3^S211^ and CESA3^T212^ have opposing effects on CESA3 activity [73]. Similarly to CSC imaging studies performed on CESA1 phosphorylation-site mutants, the authors of this study observed that CESA3^S211A^ and CESA3^T212E^ mutants exhibited CSCs with differential bidirectional motility behavior, suggesting that this facet of *in vivo* CSC behavior could be altered in both CESA1 and CESA3. Additionally, the CESA3^S211E^ and CESA3^T212E^ mutants displayed no difference in primary root length compared to wild-type plants, but their root hair length was significantly shorter, indicating that CESA3^S211^ and CESA3^T212^ phosphorylation negatively regulates root hair elongation [73]. Overall, these observations seem to suggest that CESA phosphorylation events may play a critical role in the regulation of CSC velocities and directional distribution along MTs.

Phosphorylation sites in SCW CESAs have also been identified [70]. Through immunoprecipitation and mass spectrometry, *in vivo* phosphorylation events were identified at S180, S181, and S185 of the *Arabidopsis* CESA7 as well as at S135 of CESA4. All of these phosphorylation sites occur within the N-terminal hypervariable region of these CESA isoforms. The N-terminal region of CESA7 was recombinantly produced and incubated with soluble *Arabidopsis* stem protein extracts. Under these conditions, the CESA7 N-terminal region was rapidly phosphorylated; however, longer incubation periods resulted in an apparent loss of the phosphorylated protein. Upon further analysis, co-incubation with the proteasome inhibitor MG132 showed increased stability of the phosphorylated CESA7 N-terminus, suggesting that phosphorylation may play a role in destabilizing CESA proteins, resulting in their regulated degradation by the 26S proteasome [70]. It is also important to note that CESA7-containing CSCs have been imaged by an inducible expression system in live *Arabidopsis* seedlings [74], and these experiments revealed that SCW CSCs exhibit differential velocities during cellular development. CESA7-containing CSCs increase their speeds during the mid-stage of development but then decrease their speeds during late-stage development, suggesting that the phosphorylation events discussed above, or other unidentified regulatory events, could mediate these differential speed effects.

Light quality has also been implicated as a regulator of CSC behavior; *cesa6* mutants grown under normal light conditions exhibited only small phenotypic or cellulose deficiencies compared to wild-type. However, dark-grown *cesa6* mutants exhibited decreased cellulose contents and shorter hypocotyls, suggesting that CESA6 is critical for cellulose production in the absence of light [75]. Furthermore, dark-grown seedlings require CESA6 for normal CSC motility [75]. Decreased hypocotyl lengths of dark-grown *cesa6* mutants could be rescued by activation of the red/far-red PHYTOCHROME B [75] photoreceptor [75,76]. A 10 min red-light treatment of *cesa6* mutants increased CSC motility, particularly of CSCs containing CESA5, and recovered particle velocity of CSCs containing CESA5. Interestingly, *CESA5* is partially redundant with *CESA6* in light-grown seedlings but not in dark-grown hypocotyls, suggesting that *CESA6* has a different function than *CESA5* in dark-grown hypocotyls [37,38]. Phosphoproteomics revealed that CESA5 is phosphorylated at four serine residues within the CESA5 N-terminal domain (S122, 126, 229, and 230) that do not exist in CESA6 [75]. These sites were mutated to phosphonull and phosphomimic residues and examined for hypocotyl growth recovery under the control of the *CESA6* promoter. CESA5 phosphonull mutants did not recover the dark-grown hypocotyl growth phenotype of the *cesa6* mutant, whereas the CESA5 phosphomimic mutants partially restored this phenotypic defect [75]. Interestingly, in dark-grown seedlings, CESA5 phosphomimic mutants increased the CSC velocity compared to CESA5 phosphonull mutants in the *cesa6* mutant background. In addition, CESA5 phosphonull mutants inhibited an increase in CSC velocity in light-grown seedlings, suggesting that CESA5 phosphorylation is crucial for proper function of CESA5. These observations suggest a phosphorylation-dependent role for CESA5 in the red-light-dependent regulation of the CSC velocity [75].

### 3.2. Brassinosteroid Regulation of the CSC and BIN2 Phoshorylation of CESA1

Although some studies have begun to address the effects of CESA phosphorylation on CSC dynamics and overall plant growth, the protein kinases that phosphorylate these residues have not been experimentally identified. Identifying the protein kinases responsible for CSC phosphoregulation is crucial for understanding the upstream developmental or environmental processes that regulate cellulose biosynthesis *in vivo*.

Recently, the BRASSINOSTEROID INSENSITIVE 2 (BIN2) [71] protein kinase was demonstrated to directly phosphorylate and negatively regulate *Arabidopsis* CESA1 [71]. Brassinosteroids (BRs) are a class of phytohormones that regulate plant growth and developmental processes, such as cell expansion and cell division [71,77,78,79,80]. BIN2 is a protein kinase that serves as a key regulator of BR signaling, and this kinase is regulated by the presence of BRs [77,81]. Mutations leading to increased BIN2 activity (*bin2-1*) or decreased BR biosynthesis negatively impacted overall crystalline cellulose content and CSC motility in the mutant seedlings compared to wild-type *Arabidopsis*, indicating that mutations in the BR signaling pathway negatively impact overall cellulose synthesis [71]. Synthetic peptides of experimentally supported phosphorylation sites on *Arabidopsis* CESA1, CESA3, CESA5, and KOR1 were subjected to *in vitro* kinase assays to identify possible BIN2 phosphorylation substrates within the CSC. BIN2 was demonstrated to phosphorylate a phosphoprimed synthetic peptide containing a phosphoserine residue corresponding to CESA1^S162^, and the cognate BIN2 phosphorylation site was mapped to CESA1^T157^. CESA1 mutants expressing the CESA1^T157A^ phosphonull mutant exhibited 20–40% longer dark-grown hypocotyls compared to control seedlings, indicating that BIN2 phosphorylation of CESA1^T157^ serves as a negative regulator of cellulose biosynthesis. Additionally, *bin2-1* seedlings expressing CESA1^T157A^ exhibited a significant increase in CSC velocity compared with control seedlings, further suggesting a negative regulatory role of BIN2 in cellulose biosynthesis [71]. This study identified the first experimentally supported protein kinase that directly phosphorylates a CESA protein and leads to negative regulation of cellulose synthase activity. Furthermore, this work highlights that cellulose biosynthesis is controlled in part by a critically important growth-promoting hormone signaling pathway.

Interestingly, BIN2 can regulate cellulose biosynthesis by directly phosphorylating CESA1, and this event is dependent on a preexisting phosphorylated serine at CESA1^S162^, an example of priming phosphorylation or sequential phosphorylation. Sequential phosphorylation sites recognized by different kinases can create logic gates for a combination of signal inputs. This logic can create an AND gate that ensures the final decision of multiphosphorylation is made only when a sufficient input from all upstream signaling kinases is present [82]. Therefore, the final output of BIN2/CESA1 may be determined by an AND gate, suggesting that at least two signal inputs are necessary for the final output that modulates CSC activity by BIN2 [71]. Alternatively, the unidentified CESA1^S162^ kinase may act in sequence with BIN2 to allow temporal or developmental information to control CSC activity. This multiplexed combination of possibilities could provide a rationale to explain the large number of phosphorylation sites observed in the CSC and a foundation for multiphosphorylation as an integration of signaling inputs that produce diverse functional outputs, such as CSC activity, stability, and subcellular trafficking.

### 3.3. The Cellulose Synthase-Like Family of Glycosyltransferases

While CESAs are known to synthesize cellulose, it is also clear that similar processive glycosyltransferases may synthesize additional plant cell wall polymers. A family of 30 cellulose synthase-like (*CSL*) genes are present in the *Arabidopsis* genome and exhibit sequence similarity to cellulose synthase proteins [83,84]. On the basis of a phylogenetic analysis of these *CSL* genes, the CSL family was divided into multiple subgroups, and, in *Arabidopsis*, there are six subgroups (CSLA, CSLB, CSLC, CSLD, CSLE, and CSLG). Additionally, some plant genomes contain CSLF and *CSLH* genes [34,82]. CSL proteins encode integral membrane glycosyltransferase family 2 proteins that are postulated to synthesize non-cellulosic cell wall polysaccharides, such as the polysaccharide backbones of hemicelluloses [84,85]. While the CSL enzymatic function is still being elucidated, CSLA, CSLC, and CSLF are the most well characterized CSL subfamilies [84,85]. CSLAs exhibit glucomannan synthase activity *in vitro*, while CSLFs have been implicated in mixed-linkage glucan biosynthesis [86,87]. For example, a CSLA *β*-mannan synthase [88] was identified by transcriptional profiling of guar seeds during galactomannan deposition. ManS was determined to be a *β*-mannan synthase because it produces a *β*-(1→4)-linked mannose-containing product that was hydrolyzed by endo-*β*-mannanase but was insensitive to both *β*-(1→3, 1→4)-glucanases and cellulases [88]. Several additional heterologously expressed *Arabidopsis* CSLAs have also demonstrated mannan synthase activity *in vitro* [89]. CSLCs are postulated to synthesize the *β*-(1→4)-glucan backbone of xyloglucan. For example, nasturtium [90] cDNA libraries derived from seeds during late development exhibited high *CSLC4* transcript abundance. During nasturtium seed maturation, large amounts of xyloglucan are produced and stored, suggesting that *CSLC4* plays a role in xyloglucan synthesis. *Pichia pastoris* heterologously expressing *TmCSLC4* and *AtCSLC4* revealed that both CSLC isoforms exhibit *β*-(1→4)-glucan synthase activity [90].

CSLDs are highly similar in terms of amino acid sequence to CESA proteins, with an average sequence identity of 45% [84,85] and the majority of this sequence identity exists within the conserved zinc finger and transmembrane domains. Unlike other CSL family members, CSLDs contain an elongated N-terminal and catalytic domains [91]. Using genetic complementation analysis, a chimeric version of the CSLD3 enzyme containing the CESA6 catalytic domain was created. CSLD3 localizes to the tip of elongating root hairs, and interestingly, CESA6 was expressed and localized to the root hair plasma membrane everywhere except for the growing tip. The CSLD3–CESA6 chimera displayed *β*-(1→4)-glucan synthase activity localized to the growing apical tip of directionally elongating root hairs and rescued the root hair defects of the *csld3* mutant [92], suggesting that CSLDs may play a role in glucan biosynthesis. CSLD loss-of-function mutants have implicated this class of enzymes in anisotropic expansion of tip-growing cells. For example, *csld3* and *csld2* displayed defective or complete loss-of-root-hair biogenesis, and *csld1* and *csld4* supported roles in pollen tube growth [93]. Double mutants of the CSLDs exhibited dwarfed phenotypes, and the *csld2;csld3;csld5* triple mutant was lethal [91,94]. These observations suggest that CSLD isoforms synthesize glycan products that are essential for cell-wall structural integrity in both individual-cell and whole-plant contexts. Additionally, the observation that the CESA6 catalytic domain can be used to replace the corresponding domain in CSLD3 suggests that CSLDs likely synthesize a similar glucan product to CESAs, although this hypothesis has not been tested biochemically.

### 3.4. Evidence for CSL Phosphorylation

There is increasing evidence to support the idea that phosphorylation plays a role in regulating CSC stability, motility, and activity [70,71,72,73,75]. CSLs are similar in terms of amino acid sequence and have a similar domain architecture to CESA proteins [61,84], and genetic evidence also implicates CSLs in plant cell-wall biosynthesis. The current, generally accepted hypothesis is that CESA proteins synthesize cellulose, while CSL family members synthesize non-cellulosic wall polysaccharides [84,95]. A survey of the *Arabidopsis* phosphorylation-site database PhosPhat4.0 [96,97] indicates that phosphorylated residues are present in the N-terminal and catalytic domains of many *Arabidopsis* CSL family members, including CSLA, CSLB, CSLC, CSLD, and CSLE. Interestingly, many of the most highly supported phosphorylation sites in CSLC and CSLD isoforms are within the central catalytic domain, suggesting that these phosphorylation sites may be important for controlling catalytic activity and potentially suggesting that post-translational phosphorylation may also regulate CSL function during cell-wall polysaccharide biosynthesis (Supplementary Table S2).

To date, the phosphorylation of CSL proteins has not been thoroughly investigated, but there is evidence to suggest that CSLDs are regulated by other post-translational modifications. CSLD2, CSLD3, and CSLD5 participate in cell-wall biosynthesis during cytokinesis [91], and *csld5* mutants display cell-division defects, suggesting a specialized role in cytokinesis. The CSLD5 promoter contains consensus sequences for the transcription factors MYB3R and MYB3R4, which are key transcriptional regulators of the G2/M cell-cycle phase transition, and CSLD5 is not expressed during early S-phase cellular development, suggesting a specialized role for CSLD5 in cell-plate formation. CSLD5 is rapidly degraded during the termination of the cell cycle, and rapid destabilization of CSLD5 was alleviated by incubation with MG132, an inhibitor of the 26S proteasome, suggesting that CSLD5 is regulated by post-translational ubiquitination [91]. Indeed, further immunoprecipitation experiments revealed that CSLD5 is ubiquitinated *in vivo*. CSLD5 contains several phosphoserine sites within its N-terminal region, and there is substantial cross-talk between ubiquitination and phosphorylation, suggesting that CSLD5 may be regulated by both of these post-translational processes. For example, the destabilization of CSLD5 could be mediated by phosphorylation of the serine residues in the N-terminal region of CSLD5, resulting in the recruitment of cognate *E*3 ubiquitin ligases that catalyze CSLD5 ubiquitination. Overall, these observations suggest that the post-translational regulation of CSL proteins merits more targeted investigation.

## 4. Receptor Kinases Implicated in Cellulose Biosynthesis

While the function of individual CESA phosphorylation sites is being elucidated, the protein kinases that regulate the phosphorylation status of these sites remain largely unclear. However, a handful of protein kinases have been implicated in aspects of cell-wall biosynthesis and, therefore, may serve as reasonable candidates for future investigation. Plants have developed a broad repertoire of plasma membrane-localized receptor kinases (RKs) that integrate extracellular inputs, transduce information into the cell, and rewrite cellular responses. The *Arabidopsis* genome contains approximately 600 RKs, all of which contain a ligand-binding extracellular domain, a single transmembrane domain, and a cytoplasmic kinase domain, which carries out signal transduction by phosphorylating target proteins [98]. The cell wall represents the frontier of cellular–environmental interactions and coordinates cell-wall deposition and remodeling to facilitate plant survival in changing environments [24]. Furthermore, plant cells possess a cell-wall integrity (CWI) [32] surveillance system that monitors the integrity of the cell wall, although few concrete molecular components of CWI signaling have been identified. Even when plants are not challenged by environmental stimuli, they may trigger defense responses once the deposition of cellulose and other polysaccharides is compromised [32,99,100]. Therefore, CWI-sensing protein kinases represent attractive potential protein kinases that may phosphorylate cellulose synthases or other CSL proteins in response to compromised CWI.

The first detailed cell-wall sensing candidate protein kinase to be implicated in cellulose biosynthesis was THESEUS1 (THE1), a RK from the *Catharanthus roseus* RLK (CrRLK)-like protein family. *THE1* was isolated from a *cesa6^prc1^* suppressor screen [101], and *the1* loss-of-function mutants did not rescue the cellulose biosynthesis defects of *cesa6^prc1^* but partially restored the reduced hypocotyl elongation phenotype of *cesa6^prc1^* mutants, suggesting that THE1 plays a role in CWI sensing [101]. Although the ligand of THE1 has not been identified, another CWI sensing RK, *LRR-RK MALE DISCOVERER1-INTERACTING RECEPTOR-LIKE KINASE2* (*MIK2*) [102], shares certain overlapping functions with THE1. MIK2 was identified from an assay screening for altered transcriptional responses to isoxaben, a cellulose biosynthesis inhibitor [102]. Interestingly, MIK2 regulates root growth twisting in a THE1- and CESA6-dependent manner, suggesting that it may act upstream from THE1 by an unidentified mechanism and that the root-twisting defects could be a result of altered CSC function *in vivo*. *FERONIA* (*FER*) is another CrRLK that is postulated to monitor CWI by directly interacting with or sensing cell-wall components [103,104]. FER has been implicated in many physiological processes, such as pollen tube perception [105], responses to salinity stress [104], and various hormone signaling pathways [106,107]. Wild-type *Arabidopsis* seedlings experience a biphasic spike in Ca^2+^, depending on which side of the seedling is undergoing mechanical stretching, in response to bending-induced mechanical stress [103]; *fer* loss-of-function mutants only undergo a single initial spike in Ca^2+^ upon bending, indicating that FER is required for mechanical signal transduction [103]. FER was also recently implicated in responses to salt stress in the *Arabidopsis* root. This study demonstrated that sodium chloride treatment applied to *fer* mutant roots led to decreased root elongation compared to wild-type controls. This phenotype correlated with an increased number of *fer* root cells that burst upon recovery from salinity stress. It has been commonly suggested that the FER extracellular domain may interact with cell wall polysaccharides, particularly pectic homogalacturonan–Ca^2+^ complexes, and the authors of this study provide evidence to suggest that salt treatment disrupts the structure of the pectic network [104]. The fact that *fer* mutants are hypersensitive to these treatments may suggest that FER and receptors of the CrRLK family perceive pectic polysaccharides in the cell wall and respond to their structural disruption. Finally, the wall-associated kinases (WAKs) have long been implicated in cell-wall signaling, but their precise function remains unclear. WAKs represent a large family of receptor-like kinases that were originally identified as transmembrane proteins containing a Ser/Thr kinase domain and a variable extracellular domain that are tightly associated with cell walls [108]. WAKs are expressed in many different tissues, including at organ junctions, in roots and shoots, and in mature leaves, and have been demonstrated to interact with cell-wall pectins in a Ca^2+^-dependent manner [108,109]. Genetic analyses of specific WAK mutants have implicated these protein kinases in cell expansion, responses to pathogens, and cellular differentiation [109,110,111,112]; however, the precise mechanisms of WAK signal transduction have remained largely elusive. In light of these observations, it is important to note that a recent quantitative proteomic study has begun to elucidate the molecular signaling targets downstream from WAK receptors after stimulation with pectic fragments [113]. Among these targets are a variety of other protein kinases and subcellular trafficking proteins that could potentially be regulated in response to cell-wall damage. These observations suggest that THE1, MIK2, FER, other CrRLKs, and WAK proteins are involved in CWI sensing and may represent direct or indirect regulators of cellulose biosynthesis.

Two leucine-rich repeat RKs, FEI1 and FEI2, have also been implicated in cellulose biosynthesis [99]; *fei1;fei2* double mutants display radial root swelling under high sucrose conditions, indicating a defect in anisotropic cell expansion. Both single and double mutants display no obvious phenotypic changes compared to wild-type when grown under normal sucrose conditions. The *fei1;fei2* double mutant also displayed reduced cellulose content, suggesting a role for these RKs in cellulose biosynthesis [114]. Mutants in the putative cell-surface adhesion protein SALT OVERLY SENSITIVE5 (SOS5) showed parallel mutant defects with the *fei1;fei2* double mutants described above. SOS5, FEI1, and FEI2 have all been shown to act in a singular pathway involving the plant growth hormone ethylene and CWI signaling [115], suggesting that SOS5 may interact with these receptors as a ligand or as a coreceptor. Inhibition of the ethylene biosynthesis pathway by treatment with *α*-aminoisobutyric acid (AIB), a competitive inhibitor of ACC synthase, reverted the *fei1;fei2* double-mutant root-growth morphology. Conversely, treatment with AIB did not affect the *fei1;fei2* double-mutant hypocotyl phenotype [114]. These data suggest that FEI1 and FEI2 play a role in regulating cell-wall architecture by mediating signaling pathways, such as the ethylene biosynthesis pathway, in response to extracellular signals that are currently unclear.

## 5. Conclusions

The synthesis of new cell-wall polysaccharide materials is a requirement for plant growth and likely places a heavy metabolic demand on plant tissues, suggesting that cell wall polysaccharide biosynthesis should be tightly regulated to respond to changes in environmental, developmental, or nutrient-availability status. The post-translational phosphorylation of proteins comprising the CSC indicates the emerging complexity of regulatory inputs and biochemical outputs that control cellulose biosynthesis, and these observations may lead to a paradigm for understanding the post-translational control of CSL proteins implicated in the synthesis of non-cellulosic cell wall polysaccharides. It is currently unclear why so many post-translational phosphorylation events exist within CSC or CSL proteins. However, these observations could be explained if these proteins differentially contribute to polysaccharide synthesis activity and signaling perception under changing cellular and environmental conditions. This scenario would afford several advantages towards regulating CSC and CSL behavior *in vivo*. One of these is the providing of a sequence of regulatory outputs. If each subunit could perceive non-overlapping signaling inputs from the cell, then CSCs containing different CESAs could receive multiple developmental and environmental inputs and produce one or more outputs at the same time. Multiple factors can affect CSC activity *in vivo*, such as abiotic and biotic stresses [24], temperature [116], nutrition availability, and the biological clock [30,117]. In a field setting, many of these factors occur simultaneously, and thus the mechanism described above could explain how plants integrate so many inputs and make decisions towards regulating CSC activity *in vivo*.

Compared to the overall 70–80% amino acid identity across the full-length protein, the N-terminal regions preceding the first transmembrane domain of CESAs and the hypervariable region within the central catalytic loop share merely a 40% amino acid conservation among CESA isoforms. These two regions represent the CESA hypervariable regions, and nearly all of the phosphorylation sites that affect protein stability, catalytic activity, and motility are present within these two motifs. These observations could suggest that post-translational phosphorylation dynamically regulates CSCs by coordination of multiphosphorylation events in response to cellular and environmental conditions. Hypervariable sequence diversity is the foundation of functional diversification, and the less conserved CESA N-terminal and catalytic region may contribute to this functional or regulatory divergence. 

In addition, CESA proteins have a high sequence identity with CSL family proteins, particularly proteins in the CSLD subfamily, with the highest sequence conservation existing within the transmembrane domains. Because of their similar domain architecture, it is tempting to suggest that understanding the basic roles of protein phosphorylation sites within CESA domains may functionally inform the regulatory consequences of similar phosphorylation sites in CSL domains. Overall, the phosphoregulation of plant cell wall biosynthesis is emerging as an important regulatory control point that responds to developmental and environmental conditions. Future work to understand the functional consequences of the myriad of CSC and CSL-associated phosphorylation sites as well as the protein kinases and upstream stimuli that control these phosphorylation events will provide critical information to understand how plant cell wall biosynthesis is controlled and coordinated.

## Supplementary Materials

The following are available online at http://www.mdpi.com/2223-7747/7/3/52/s1. Table S1: Experimentally supported phosphorylation sites identified on CESA and CSC-associated proteins, Table S2: Experimentally supported phosphorylation residues on Arabidopsis CSL family proteins.
