# Supplementary material for: Phosphoregulation of the Plant Cellulose Synthase Complex and Cellulose Synthase-Like Proteins
Source: Plants (Basel). 2018 Jun 29;7(3):52. doi: 10.3390/plants7030052 (PMC6161211; doi:10.3390/plants7030052)
Supplement: Supplementary file 1 [file plants-07-00052-s001.pdf]

Supplemental Table S1. Experimentally supported phosphorylation sites identified on CESA and CSC-associated proteins.

Phosphorylation Site [1]	Protein Domain Location [2]	# of Peptides Observed [3]	Regulatory Function
CESA1 S24	N-term	1	
CESA1 S134	N-term	1	
CESA1 S152	N-term	2	
CESA1 T157	N-term	1	Activity
CESA1 S162	N-term	3	Bi-directional motility
CESA1 T165	N-term	2	Bi-directional motility
CESA1 T166	N-term	--	Bi-directional motility
CESA1 S167	N-term	10	Bi-directional motility
CESA1 S179	N-term	2	
CESA1 S180	N-term	2	
CESA1 T243	N-term	1	
CESA1 S686	Catalytic	4	Bi-directional motility
CESA1 S688	Catalytic	4	Bi-directional motility
CESA2 S11	N-term	4	
CESA3 S3	N-term	3	
CESA3 T7	N-term	1	
CESA3 S130	N-term	1	
CESA3 S139	N-term	1	
CESA3 T143	N-term	2	
CESA3 S144	N-term	3	
CESA3 S151	N-term	37	
CESA3 Y169	N-term	2	
CESA3 S170	N-term	1	
CESA3 S171	N-term	2	
CESA3 S176	N-term	20	
CESA3 S211	N-term	7	Bi-directional motility
CESA3 T212	N-term	5	Bi-directional motility
CESA3 S216	N-term	17	
CESA3 S226	N-term	2	
CESA3 T227	N-term	1	
CESA3 S249	N-term	1	
CESA3 S671	Catalytic	1	
CESA4 S135	N-term	3	
CESA4 S136	N-term	1	
CESA4 S139	N-term	2	
CESA4 S374	Catalytic	1	
CESA5 S11	N-term	4	
CESA5 S122	N-term	3	Activity
CESA5 T124	N-term	1	
CESA5 S126	N-term	4	Activity
CESA5 S229	N-term	2	Activity
CESA5 S230	N-term	1	Activity
CESA6 S11	N-term	4	
CESA7 S180	N-term	--	Subunit stability
CESA7 S181	N-term	1	Subunit Stability
CESA7 S185	N-term	2	Subunit Stability
KOR1 T20		3	
KOR1 S25		4	
KOR1 S37		5	
CSI1 S20		1	
CSI1 S33		1	
CSI1 T37		2	
CSI1 S40		1	
CSI1 T42		1	
CSI1 T50		3	

CSI1 T51		2
CSI1 S52		3
CSI1 T59		3
CSI1 S62		1
CSI1 S1403		1
CC1 T10	N-term	3
CC1 S11	N-term	4
CC1 S15	N-term	10
CC1 S16	N-term	25
CC1 S20	N-term	31
CC1 S30	N-term	15
CC1 S32	N-term	2
CC1 S35	N-term	2
CC1 T48	N-term	2
CC1 S56	N-term	2
CC1 S62	N-term	1
CC1 S77	N-term	1
CC1 S79	N-term	1
CC1 S84	N-term	1
CC1 S93	N-term	2
CC2 T10	N-term	1
CC2 S15	N-term	1
CC2 S16	N-term	4
CC2 T18	N-term	1
CC2 S20	N-term	4
CC2 S30	N-term	1

[1] The common protein name and experimentally supported phosphorylation site for members of the CSC is shown. The corresponding AGI numbers are CESA1 (AT4G32410), CESA2 (AT4G39350), CESA3 (AT5G05170), CESA4 (AT5G44030), CESA5 (AT5G09870), CESA6 (AT5G64740), CESA7 (AT5G17420), KOR1 (AT5G49720), CSI1 (AT2G22125), CC1 (AT1G45688), and CC2 (AT5G42860).
[2] Domain location of each phosphorylation site within the corresponding protein
[3] The number of spectral occurrences supporting each phosphorylation site is shown according to spectral counts in PhosPhat 4.0 (http://phosphat.uni-hohenheim.de/)

Supplemental Table S2. Experimentally supported phosphorylation residues on Arabidopsis CSL family proteins.

**Phosphorylation Site** [1]	**# of Peptides Observed** [2]
CSLA1 Y232	2
CSLA1 S236	2
CSLA2 Y162	1
CSLA2 S317	1
CSLA2 T318	1
CSLB3 T610	2
CSLC4 T558	1
CSLC4 S561	6
CSLC4 S562	6
CSLC4 S564	7
CSLC4 S581	40
CSLC5 S590	1
CSLC5 S592	2
CSLC5 S614	7
CSLC6 S60	1
CSLC6 S570	1
CSLC6 Y572	1
CSLC6 T577	1
CSLC6 S606	1
CSLC6 S607	1
CSLC6 S608	11

CSLC8 S509	2
CSLC12 S604	1
CSLD1 S110	1
CSLD2 S10	1
CSLD2 S12	2
CSLD2 S15	2
CSLD2 T225	2
CSLD2 T398	3
CSLD2 T401	1
CSLD2 S404	4
CSLD2 Y410	4
CSLD3 S12	5
CSLD3 S15	2
CSLD3 T16	1
CSLD3 S18	1
CSLD3 T35	1
CSLD3 T226	2
CSLD3 S755	43
CSLD4 T502	1
CSLD5 S32	1
CSLD5 S45	2
CSLD5 S56	1
CSLD6 Y306	1
CSLD6 Y311	1
CSLE1 Y189	1
CSLE1 S191	1
CSLE1 S192	1

[1] The common name and experimentally supported phosphorylation site for each identified CSL isoform is shown (https://www.arabidopsis.org/).

[2] The corresponding number of observed phosphopeptides supporting each phosphorylation site in the PhosPhat 4.0 database is shown (http://phosphat.uni-hohenheim.de/).
